# Digitoflavone Inhibits IκBα Kinase and Enhances Apoptosis Induced by TNFα through Downregulation of Expression of Nuclear Factor κB-Regulated Gene Products in Human Pancreatic Cancer Cells

**DOI:** 10.1371/journal.pone.0077126

**Published:** 2013-10-11

**Authors:** Xueting Cai, Wuguang Lu, Yang Yang, Jie Yang, Juan Ye, Zhenhua Gu, Chunping Hu, Xiaoning Wang, Peng Cao

**Affiliations:** 1 Jiangsu Branch of China Academy of Chinese Medical Sciences, Nanjing, China; 2 Laboratory of Cellular and Molecular Biology, Jiangsu Province Academy of Traditional Chinese Medicine, Nanjing, China; Temple University School of Medicine, United States of America

## Abstract

Tumor necrosis factor-α (TNFα) activates both cell death and cell survival pathways. The activation of survival pathway renders most cancer cells resistant to TNF-induced cytotoxicity. We found that pretreatment with digitoflavone, a plant flavonoid, greatly sensitized TNFα-induced apoptotic cell death in several human pancreatic cancer cells. In search of the molecular basis of the sensitization effect of digitoflavone, digitoflavone was found to inhibit TNFα-induced activation of nuclear transcription factor-kappa B (NF-κB) which is the main survival factor in TNFα signaling. NF-κB suppression occurred through inhibition of IκBα kinase activation, IκBα phosphorylation, IκBα degradation, and NF-κB nuclear translocation. This inhibition correlated with suppression of NF-κB-dependent genes involved in antiapoptosis (mcl-1, bcl-2, bcl-xl, c-iap1, c-iap2, flip, and survivin), proliferation (c-myc, cyclin d1), and angiogenesis (vegf, cox-2, and mmp-9). In addition, digitoflavone can activate JNK through inhibition of NF-κB signaling, provide a continuous blockade of the feed-back inhibitory mechanism by JNK-induced NF-κB activation. This study found a novel function of digitoflavone and enhanced the value of digitoflavone as an anticancer agent.

## Introduction

Pancreatic cancer is the fourth leading cause of death in cancer patients in the U.S. and is a global cancer treatment problem [1]. Traditional treatment modalities for unresectable pancreatic cancer include radiation alone, chemotherapy alone, or combined chemoradiation. However, one-year and five-year survival rates are only <15% and 5%, respectively [2], [3]. The principal drug currently used in the treatment of patients who have pancreatic cancer is gemcitabine, which has an objective response rate of only 5% [4]. Chemoresistance of tumor cells is apparently the major cause of failure of conventional chemotherapy in the treatment of pancreatic cancer. Nuclear factor-κB (NF-κB) is one of the contributing factors involved in resistance to chemotherapy [5], [6]. More than 90% of pancreatic cancer cells harbor mutated K-ras [7], and NF-κB is a downstream effector of this oncogenic Ras [8], [9], [10]. NF-κB is constitutively activated in primary pancreatic adenocarcinoma and pancreatic cancer cell lines [8], and downregulated NF-κB forms the biological rationale for effective management of patients with pancreatic carcinoma by using a nontoxic phytochemical [11]. Furthermore, inflammation is suggested to be a critical component of pancreatic cancer [12], and NF-κB activation is essential in the inflammatory process [13]. Thus, the development of compounds that target NF-κB is proposed as an approach for the treatment of patients with pancreatic cancer [6], [14], [15].

Nuclear transcription factor-kappa B (NF-κB) is critically important for tumor cell survival, growth, angiogenesis, and metastasis. Under normal conditions, NF-κB, which consists of p50, p65, and IκBα, is localized in the cytoplasm. However, when activated, this transcription factor translocates to the nucleus. In response to an activation signal, the inhibitory IκBα subunit undergoes phosphorylation, ubiquitination, and degradation, thus exposing nuclear localization signals on the p50-p65 heterodimer. The p65 is then phosphorylated, which leads to its nuclear translocation and binding to a specific sequence in DNA, which in turn results in gene transcription [16], [17]. NF-κB has been shown to regulate the expression of a number of genes, the products of which are involved in tumorigenesis [17], [18], [19], [20], [21]. These include antiapoptotic genes (e.g., ciap, survivin, traf, cflip, bfl-1, bcl-2, and bcl-xl), inflammatory genes (cox-2, mmp-9, and vegf), and genes which encode adhesion molecules, chemokines, and cell cycle regulatory genes (e.g., cyclin d1 and c-myc). Thus, agents that suppress NF-κB activation have therapeutic potential for pancreatic carcinoma [22], [23], [24], [25], [26], [27], [28].

Digitoflavone (Dig) is a common flavonoid that is present in many types of plants such as fruits, vegetables, and medicinal herbs. Plants rich in digitoflavone have been used in Chinese traditional medicine for treating various diseases such as hypertension, inflammatory disorders, and cancer. Digitoflavone’s anticancer property is associated with the induction of apoptosis and inhibition of cell proliferation, metastasis, and angiogenesis [29]. Digitoflavone significantly sensitized TNFα-induced apoptosis in a number of human pancreatic cancer cell lines, an effect which was discovered for the first time by this study. Such sensitization is closely associated with digitoflavone’s inhibitory effect on NF-κB activation, which downregulated some key antiapoptotic genes such as c-iap1 and vegf. Digitoflavone could activate JNK, a critical process in the sensitization of digitoflavone on TNFα-induced apoptosis. Data from this study advanced our understanding of the molecular mechanism involved in the anticancer activity of digitoflavone.

## Materials and Methods

### 2.1 Materials

Digitoflavone was purchased from Nanjing TCM Institute of Chinese Materia Medica, China. It was dissolved in dimethyl sulfoxide (DMSO) as a 20 mmol/L stock solution and stored at −20°C. *Escherichia coli*-derived human tumor necrosis factor-α (TNF-α), which is suitable for cell culture, was obtained from Sigma, Inc. Trypsin and MTT were obtained from Sigma, USA. Lysis buffer was purchased from Beyotime, China. Antibodies (caspase-3, caspase-8, goat anti-mouse IgG-HRP and goat anti-rabbit IgG-HRP) were obtained from Santa Cruz, USA. Antibodies Cycle D1, COX-2, MMP-9, VEGF, Mcl-1, Bcl-2, Bcl-X_L_, c-IAP1, c-IAP2, FLIP, Survivin, PARP, IKKα/β, p-IKKα, p-IKKβ, IκBα, p- IκBα, JNK, and p-JNK were purchased from Cell Signaling Technology, USA. Monoclonal mouse anti-glyceraldehyde-3-phosphate dehydrogenase (GAPDH) was obtained from Kangchen, China. The p65 expression vector, pCMV-p65, was a kind gift from Dr Fang Wang, Harvard Medical School, Boston.

### 2.2 Cell Culture

Human pancreatic cell lines PANC-1, CoLo-357, and BxPC-3 were purchased from the Cell Bank of Shanghai Institute of Biochemistry and Cell Biology. Cells were cultured in DMEM medium (for PANC-1, CoLo-357 cells) or RPMI-1640 (for BxPC-3 cells) supplemented with 10% fetal bovine serum (FBS), 100 U/mL penicillin and 100 µg/mL streptomycin (all available from Invitrogen, Grand Island, NY, USA). All cultures were maintained in a humidified environment with 5% CO_2_ at 37°C.

### 2.3 Annexin-V/PI Double Staining Assay

Pancreatic cancer cells were treated with digitoflavone (40 µM), TNFα (20 ng/mL) alone or together at 37°C for 24 h. The cells were then harvested, washed, and resuspended with PBS. Apoptotic cells were determined by using an FITC Annexin V Apoptosis Detection Kit (BD Biosciences, USA) according to the manufacturer’s protocol. The cells were washed briefly and subsequently incubated for 15 min in 100 µL of 1 × binding buffer, which contains 5 µL of Annexin V-FITC and 5 µL of PI, in the dark at room temperature. Afterward, apoptosis was analyzed by FACScan laser flow cytometer (FACSCalibur, Becton Dickinson, USA). The data were analyzed using the software CELLQuest.

### 2.4 NF-κB Luciferase Reporter Assay

PANC-1 cells were transiently transfected with the NF-κB dependent firefly luciferase reporter construct and constitutively expressing Renilla luciferase construct (40∶1) using the Lipofectamine 2000 according to the manufacturer’s protocol (Invitrogen, USA). Firefly luciferase activity was determined and normalized to the control Renilla level, using the Dual-Luciferase Reporter Assay System (Promega USA).

### 2.5 NF-κB Activation

The DNA binding activity of NF-κB was determined by electrophoretic mobility shift assay (EMSA) followed instructions of LightShift Chemiluminescent EMSA Kit (Pierce, USA). Nuclear extracts were prepared using NE-PER Nuclear and Cytoplasmic Extraction Kit (Pierce, USA), according to the manufacturer’s instructions. Nuclear extracts were incubated with biotin end-labeled, double-stranded NF-κB oligonucleotide (5′-AGTTGAGGGGACTTTCCCAGG-3′) or Oct-1 oligonucleotide (5′-TGTCGAATGCAAATCACTAGA A-3′) (Beyotime, China) for 20 min at room temperature. The DNA-protein complex formed was separated on 5% native polyacrylamide gel. The DNA was then rapidly transferred to a positive nylon membrane, UV crosslinked, probed with streptavidin-HRP conjugate, incubated with substrate and exposed to X-ray film.

### 2.6 IκBα Degradation and Phosphorylation

To determine the effect of digitoflavone on TNFα-dependent IκBα degradation and phosphorylation, cytoplasmic extracts were prepared as described previously [30] from pancreatic cancer cells pretreated with digitoflavone for 7 h and then exposed to 20 ng/mL TNFα for 5, 10, and 20 min. The extracts were then resolved on 13% SDS-polyacrylamide gels. After electrophoresis, the proteins were electrotransferred to PVDF membranes (Millipore, USA), probed with antibodies against IκBα and phosphorylated IκBα, and detected by using chemiluminescence (Luminata Crescendo Western HRP substrate, Millipore, USA).

### 2.7 Binding Potency of Digitoflavone to the ATP Binding Site of IKK

To determine the binding potency of digitoflavone to the ATP binding site of IKK, we performed kinase binding assay by KINOMEscan (LeadHunter Discovery Services). Briefly, kinase-tagged T7 phage strains were prepared in an *E. coli* host derived from the BL21 strain. *E. coli* were grown to log-phase and infected with T7 phage and incubated with shaking at 32°C until lysis. The lysates were centrifuged and filtered to remove cell debris. The remaining kinases were produced in HEK-293 cells and subsequently tagged with DNA for qPCR detection. Streptavidin-coated magnetic beads were treated with biotinylated small molecule ligands for 30 minutes at room temperature to generate affinity resins for kinase assays. The liganded beads were blocked with excess biotin and washed with blocking buffer (SeaBlock (Pierce), 1% BSA, 0.05% Tween 20, 1 mM DTT) to remove unbound ligand and to reduce nonspecific binding. Binding reactions were assembled by combining kinases, liganded affinity beads, and test compounds in 1× binding buffer (20% SeaBlock, 0.17× PBS, 0.05% Tween 20, 6 mM DTT). All reactions were performed in polystyrene 96-well plates in a final volume of 0.135 ml. The assay plates were incubated at room temperature with shaking for 1 hour and the affinity beads were washed with wash buffer (1× PBS, 0.05% Tween 20). The beads were then re-suspended in elution buffer (1× PBS, 0.05% Tween 20, 0.5 µM non-biotinylated affinity ligand) and incubated at room temperature with shaking for 30 minutes. The kinase concentration in the eluates was measured by qPCR.

### 2.8 JNK Activation Assay

To determine the effect of digitoflavone on TNFα-induced JNK activation, Western blot was used to perform JNK assay. Briefly, cytoplasmic extracts were prepared from pancreatic cancer cells treated with 40 µM digitoflavone for 7 h and then treated with 20 ng/mL TNFα for 5, 10, and 20 min. The extracts were then resolved on 13% SDS-polyacrylamide gels and analyzed by Western blot by using an antibody against JNK and p-JNK.

### 2.9 Transfection of p65

Transfection was performed in 6-well plates using Lipofectamin 2000 reagent (Invitrogen, Paisley, UK) according to the manufacturer’s instructions. Briefly, cells were grown in 6-well plates and transfected with the appropriate vector (2 µg) the following day. After 4 h the transfection mix was removed and replaced with complete medium. Cell treatments were then carried out 24 h post-transfection as indicated.

### 2.10 NF-κB Targeted Gene Expression Analysis

To determine the effect of digitoflavone on TNFα-induced NF-κB targeted gene expression, Western blot was used to perform protein expression assay. Briefly, cytoplasmic extracts were prepared from pancreatic cancer cells untreated or pre-treated with 40 µM digitoflavone for 7 h and then treated with 20 ng/mL TNFα for 2, 4, 8, 12, and 24 h.

### 2.11 Real-Time Quantitative PCR (qPCR)

Total RNA were extracted using Trizol reagent (Invitrogen, Carlsbad, CA) according to the manufacturer’s instructions. 2 µg of total RNA was used for cDNA synthesis with random hexamer primers. qPCR was carried out using an ABI PRISM 7500 Sequence Detection System (Applied Biosystems). Reactions were performed per SYBR Green instructions (Thermo scientific, USA) in triplicate in three independent experiments. The primer sequences are provided in table 1. The ΔΔC_T_ method was used for qPCR determination. GAPDH was used as housekeeping gene to normalize the variability in expression levels.

**Table 1:** Primer sequences used for real-time quantitative PCR (5′ to 3′).

Gene	Forward primer	Reverse primer
hGAPDH	ACATCAAGAAGGTGGTGAAGCA	GTCAAAGGTGGAGGAGTGGGT
hc-Myc	CCTTGCCGCATCCACGAAA	GCGTCCTTGCTCGGGTGTT
hMMP-9	AGTCCACCCTTGTGCTCTTCC	TGCCACCCGAGTGTAACCAT
hVEGF	AGGGAAGAGGAGGAGATGA	GGCTGGGTTTGTCGGTGTT
hCOX-2	CCGAGGTGTATGTATGAGTGT	CCTTGAAGTGGGTAAGTATGT
hCyclin D1	TCCTCTCCAAAATGCCAGAG	GGCGGATTGGAAATGAACTT
hBcl-2	CTGAGTACCTGAACCGGCA	GAGAAATCAAACAGAGGCCG
hBcl-X_L_	TTCAGTGACCTGACATCCCA	CTGCTGCATTGTTCCCATAG
hMcl-1	AAAGCCTGTCTGCCAAAT	ATAAACCCACCACTCCC

### 2.12 VEGF Detection by ELISA

VEGF concentration in the conditioned medium from human pancreatic carcinoma cells was measured by using a commercially available ELISA kit (R&D Systems, Minneapolis, MN, USA). The cells (3×10^5^/well) were incubated overnight in 6-well dishes in a medium which contains 10% FBS. The media were then replaced for 24 h with serum-free media which contain digitoflavone, TNFα or digitoflavone combined with TNFα. VEGF was expressed as a picogram of VEGF protein per milliliter medium and per 10^5^ cells.

### 2.13 Statistical Analysis

The numeric data are presented as mean±s.d. from at least three sets of independent experiments. The differences among different groups were examined using a one-way ANOVA with Scheffe’s test (SPSS 11) and *P*<0.05 was considered statistically significant.

## Results

### 3.1 Digitoflavone Potentiated Apoptotic Effects of TNFα

**Figure 1:**
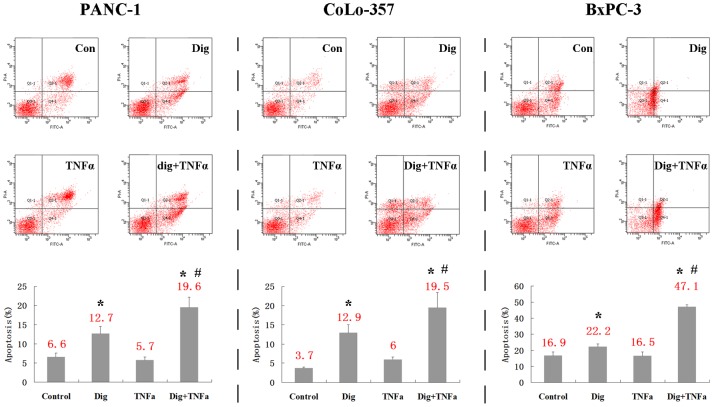
Pancreatic cancer cells were serum starved for 12α (20 ng/mL), digitoflavone (40 µM), alone or together for 24 h. Cell apoptosis was determined by Annexin V FITC Apoptosis Kit. *P<0.05 comparing to non-treated control; and #*P*<0.05 comparing to the group with TNFα only.

The effects of digitoflavone on the apoptotic effects of TNFα were examined. TNFα by itself did not induce a significant amount of apoptosis; however, when combined with digitoflavone, the cytotoxic effects of TNFα were enhanced (Fig. 1). Digitoflavone combined with TNFα increased by about 180–240% apoptosis rate than TNFα alone.

### 3.2 Digitoflavone Suppressed NF-κB-dependent Reporter Gene Expression Induced by TNFα

**Figure 2:**
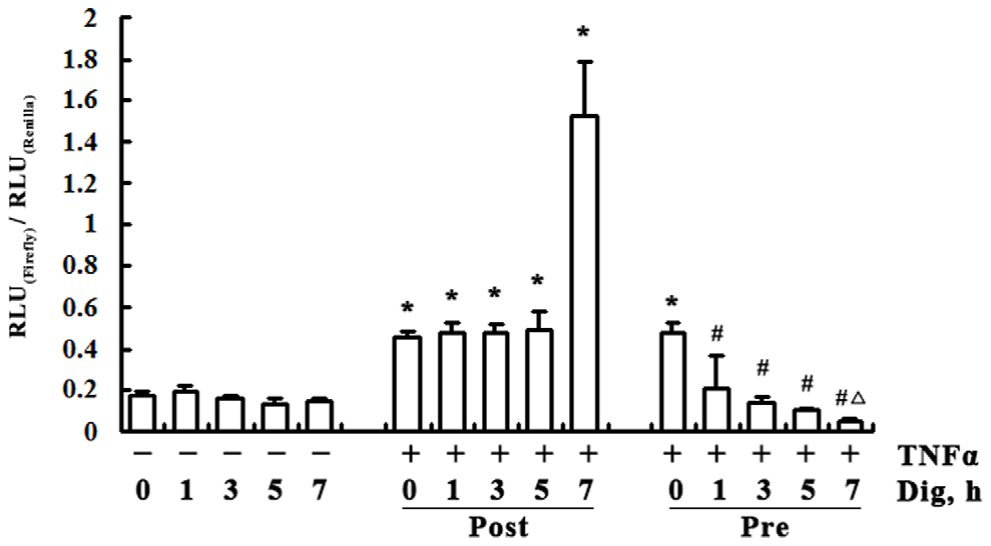
Digitoflavone inhibited TNFα-induced NF-κB transcriptional activity. PANC-1 cells were cotransfected with NF-κB dependent firefly luciferase reporter construct and constitutively expressing Renilla luciferase construct. The cells were then treated with digitoflavone (40 µM) for different times (1 h, 3 h, 5 h, 7 h). Another group cells (the pretreatment group) were treated with digitoflavone (40 µM) for different times (1 h, 3 h, 5 h, 7 h), followed by TNFα (20 ng/mL) for 2 h. The post-treatment group were treated with TNFα (20 ng/mL) for 2 h, followed by digitoflavone (40 µM) for different times (1 h, 3 h, 5 h, 7 h). Luciferase activity was expressed as fold increased over control after normalized with Renilla luciferase activity. Data are presented as means±s.d. from at least three independent experiments. *P<0.05 comparing to TNFα-nontreated control (0 h); ^#^ *P*<0.05 comparing to the group with TNFa only, and ^△^ *P*<0.05 comparing to the group TNFα-nontreated Dig-treated control (7 h).

We examined the inhibitory effect of digitoflavone on NF-κB transcriptional activity in PANC-1 cells by using the NF-κB luciferase reporter assay. As shown in Figure 2, treatment with TNFα significantly enhanced NF-κB transcriptional activity and digitoflavone pretreatment markedly suppressed the transactivation of NF-κB induced by TNFα. The reduced luciferase activity by digitoflavone may due to its direct inhibition on luciferase enzyme activity. To exclude such a possibility, digitoflavone post-treatment was conducted. Cells were first treated with TNFα (20 ng/mL) for 2 h followed by digitoflavone (40 µM) treatment for another 2 h. It is rather interesting to find that digitoflavone post-treatment failed to inhibit the transactivation of NF-κB induced by TNFα, suggesting that digitoflavone does not suppress NF-κB activation post-transcriptionally and pose no direct inhibition to luciferase enzyme activity.

### 3.3 Digitoflavone Inhibited Inducible NF-κB Activation by TNFα

**Figure 3:**
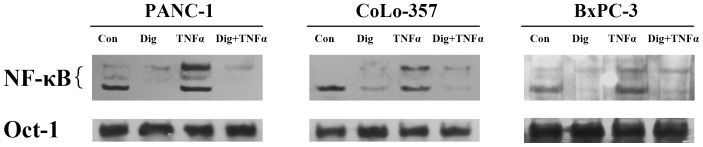
Digitoflavone inhibited inducible NF-κB activation by TNFα. Pancreatic cancer cells were pre-incubated with digitoflavone (40 µM) for 7 h and then treated with 20 ng/mL TNFα for 30 min at 37°C. Nuclear extracts were prepared and then tested for NF-κB activation by EMSA. Bottom, EMSA using an Oct-1 probe for a loading control.

TNFα is an activator of NF-κB, and the mechanism of NF-κB induction reportedly varies among different cell types. [31] Thus, we examined whether digitoflavone was effective in blocking NF-κB activation in three human pancreatic cancer cell lines. According to results, digitoflavone completely inhibited TNFα-induced NF-κB activation in all three cell lines (Fig. 3), thereby indicating that digitoflavone was effective in inhibiting TNFα-inducible NF-κB in pancreatic cancer cell lines of varying differentiation.

### 3.4 Digitoflavone Inhibited TNFα-dependent IκBα Phosphorylation and Degradation

**Figure 4:**
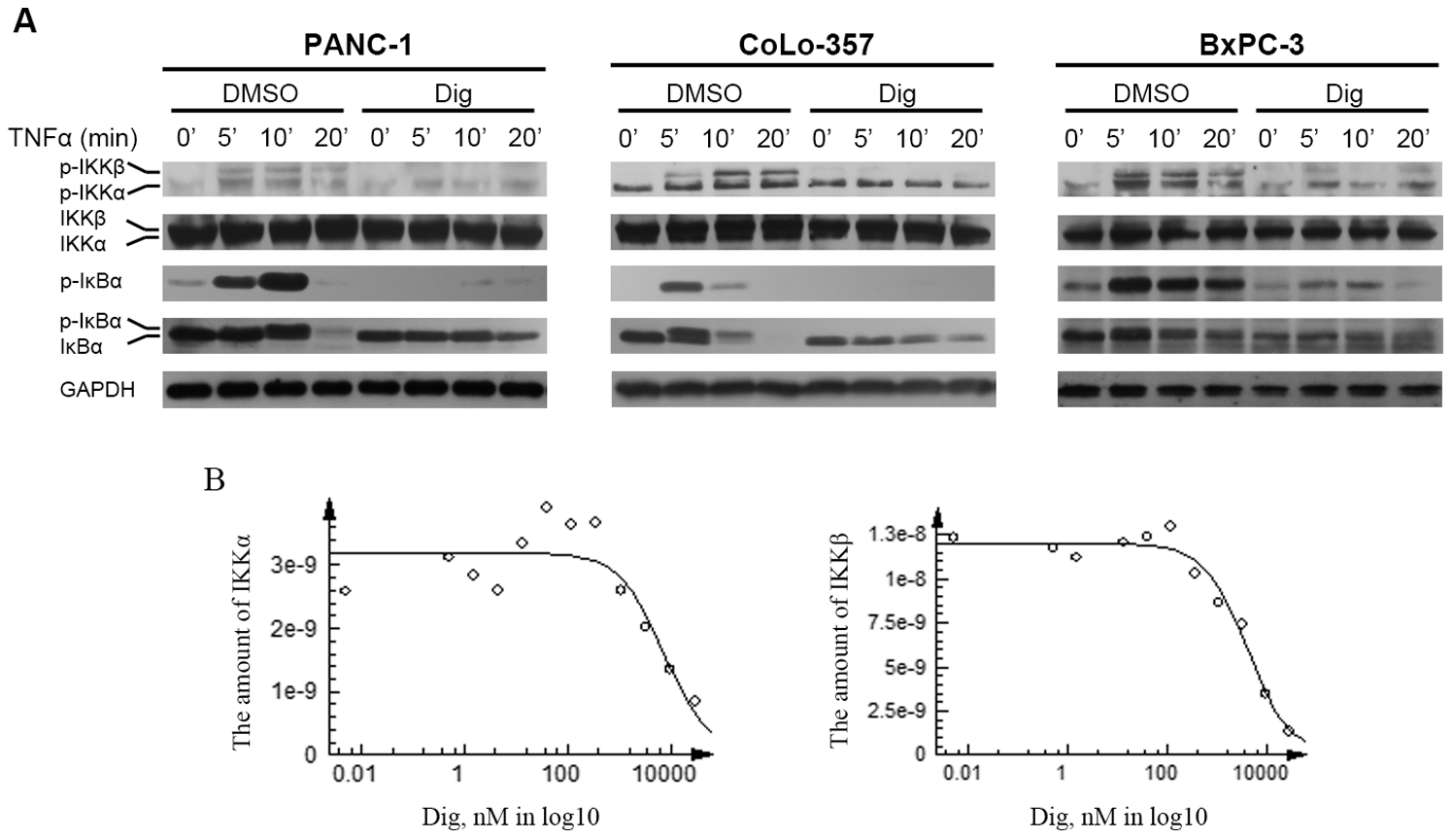
Digitoflavone inhibited TNFα-induced IKK activation. A, digitoflavone inhibited TNFα-induced phosphorylation of IKKα/β and IκBα. Pancreatic cancer cells were incubated with 40 µM digitoflavone for 7 h before exposing to TNFα for different times, and then tested for phosphorylated IKKα/β and IκBα in cytosolic fractions by Western blotting analysis. B, digitoflavone had a good binding potency to the ATP binding site of IKK, with Kds of 7.3 µM and 5.2 µM for IKKα and IKKβ respectively.

IκBα phosphorylation is required for NF-κB activation. Therefore, we aimed to determine whether digitoflavone affected TNFα-induced IκBα phosphorylation which is another condition for NF-κB translocation. According to Western blot analysis which used an antibody that detects only the serine-phosphorylated form of IκBα, digitoflavone completely suppressed TNFα-induced IκBα phosphorylation (Fig. 4A). IκBα degradation is typically required for the translocation of NF-κB to the nucleus. Therefore, we aimed to determine whether inhibition of TNFα-induced NF-κB activation by digitoflavone was due to inhibition of IκBα degradation. We found that TNFα induced IκBα degradation in control cells and digitoflavone delayed TNFα-induced IκBα degradation on PANC-1 and Colo-357 cells (Fig. 4A). On the other hand, digitoflavone pretreatment partially inhibited the expression of IκBα (time point 0′).

### 3.5 Digitoflavone Inhibited TNFα-induced IKK Activation

IKK activation is critical for TNFα-induced NF-κB activation. Digitoflavone completely suppressed TNFα-induced activation of IKK. Neither TNFα nor digitoflavone exerted any direct effect on the expression of IKK proteins (Fig. 4A). Results from KINOMEscan assay revealed that digitoflavone had a good binding potency to the ATP binding site of IKK, with Kds of 7.3 µM and 5.2 µM for IKKα and IKKβ respectively (Fig. 4B). These results demonstrated that digitoflavone very likely downregulated the expression of NF-κB-regulated gene products through inhibition of IKK.

### 3.6 Digitoflavone Could Active JNK and this Effect was Blocked by Overexpression of p65

**Figure 5:**
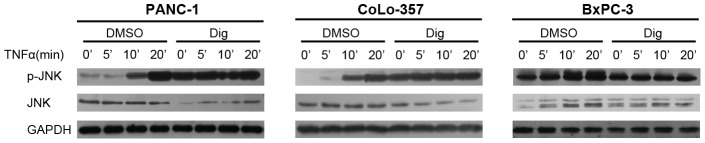
Digitoflavone could activate JNK, as well as TNFα, and the activation effect was not weakened when they used together. Pancreatic cancer cells were incubated with 40 µM digitoflavone for 7 h at 37°C, and then tested for phosphorylated JNK in cytosolic fractions by Western blotting analysis.

**Figure 6:**
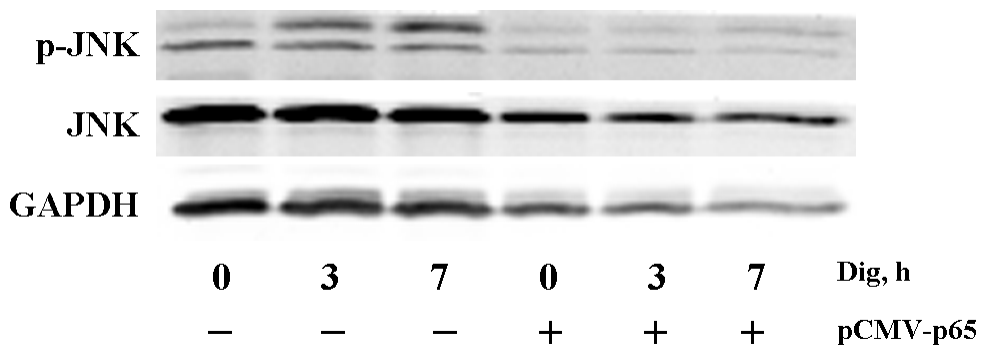
Overexpression of p65 prevented digitoflavone-induced JNK activation. PANC-1 Cells were transfected with pCMV-p65 or empty vector and treated with digitoflavone (40 µM) for 3 h or 7 h. JNK activity was determined by western blotting in whole-cell lysates.

We examined the effect of digitoflavone pretreatment on TNFα-induced JNK activation. TNFα alone caused rapid and transient JNK activation in pancreatic cancer cells, as demonstrated by increased JNK phosphorylation. Digitoflavone could activate JNK, as well as TNFα, and the activation effect was not weakened when they used together (Fig. 5). To determine the effects of overexpression of p65 on JNK activation, we transiently transfected PANC-1 cells with p65 or empty vector and assessed whole-cell lysates from digitoflavone-treated cells by western blotting analysis using an antibody that specifically recognizes the phosphorylated form of JNK. As shown in Figure 6, digitoflavone treatment resulted in sustained phosphorylation of both p46 and p54 isoforms of JNK. Overexpression of p65 blocked digitoflavone -induced JNK activation.

### 3.7 Digitoflavone Inhibited NF-κB-regulated Gene Products Expression

**Figure 7:**
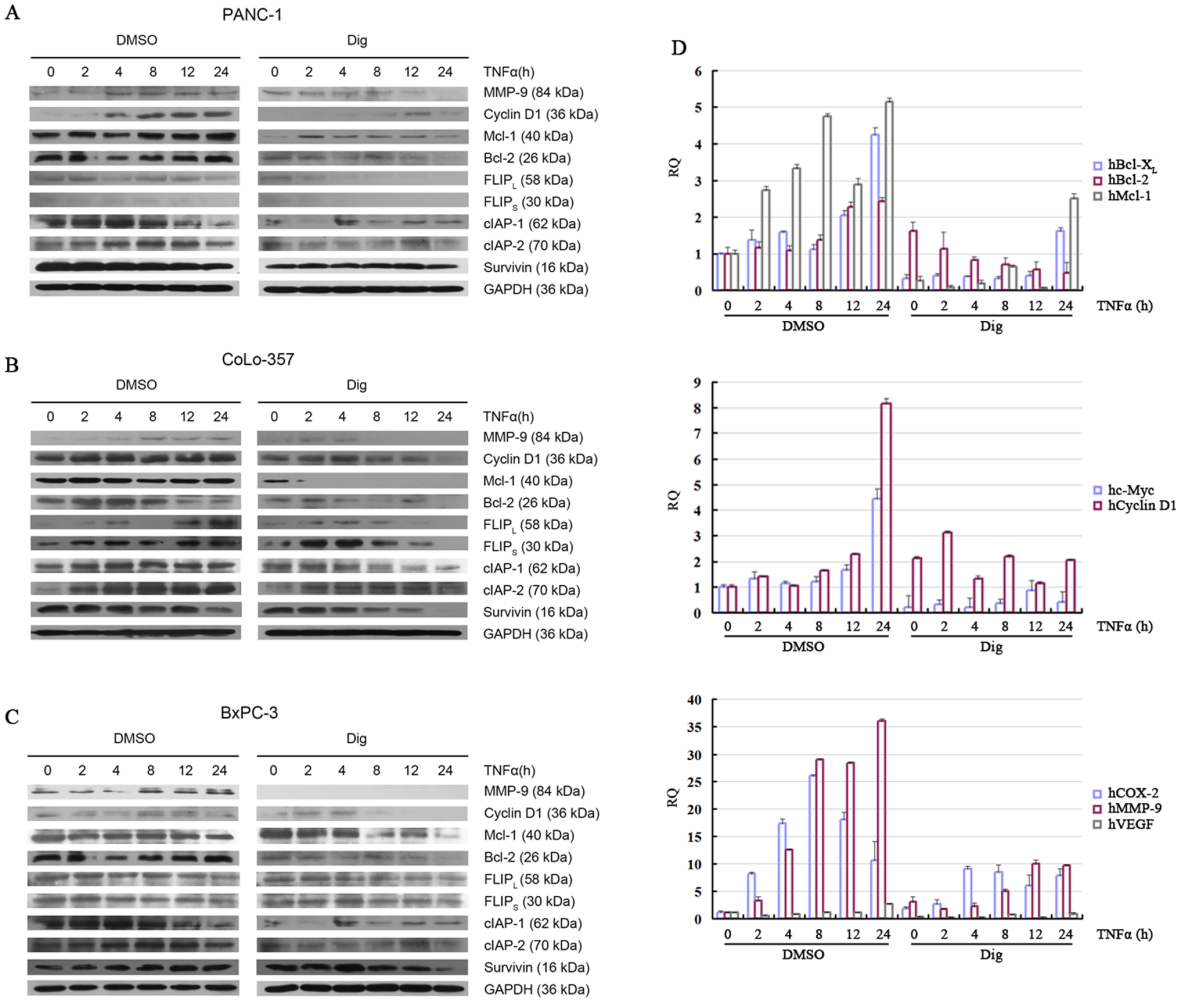
Digitoflavone inhibited expression of antiapoptosis proteins, proliferation proteins, and angiogenesis proteins induced by TNFα. Pancreatic cancer cells were left untreated or incubated with 40 µM digitoflavone for 7 h and then exposed to TNFα for different times. Whole cell extracts were prepared and analyzed by Western blotting (Fig. 7A–C) or qPCR (Fig. 7D).

COX-2, MMP-9, and vascular endothelial growth factor (VEGF) are known to be regulated by NF-κB. Cyclin D1 and c-Myc regulate cellular proliferation and are regulated by NF-κB. NF-κB upregulates the expression of a number of genes implicated in facilitating tumor cell survival, such as Mcl-1, Bcl-2, Bcl-X_L_, c-IAP1, c-IAP2, FLIP and survivin. Thus, the effect of digitoflavone on the expression of these NF-κB-regulated genes and gene products were also examined. TNFα treatment induced the expression of MMP-9, Cyclin D1, Mcl-1, Bcl-2, c-IAP1, c-IAP2, FLIP and surviving gene products, and digitoflavone abolished TNFα-induced expression of these gene products (Fig. 7A–C). Our results also indicated that digitoflavone abolished TNFα-induced mRNA level of COX-2, MMP-9, VEGF, Cyclin D1, c-Myc, Mcl-1, Bcl-2 and Bcl-X_L_ (Fig. 7D).

### 3.8 Digitoflavone Suppressed VEGF Secretion from Pancreatic Carcinoma Cells

**Figure 8:**
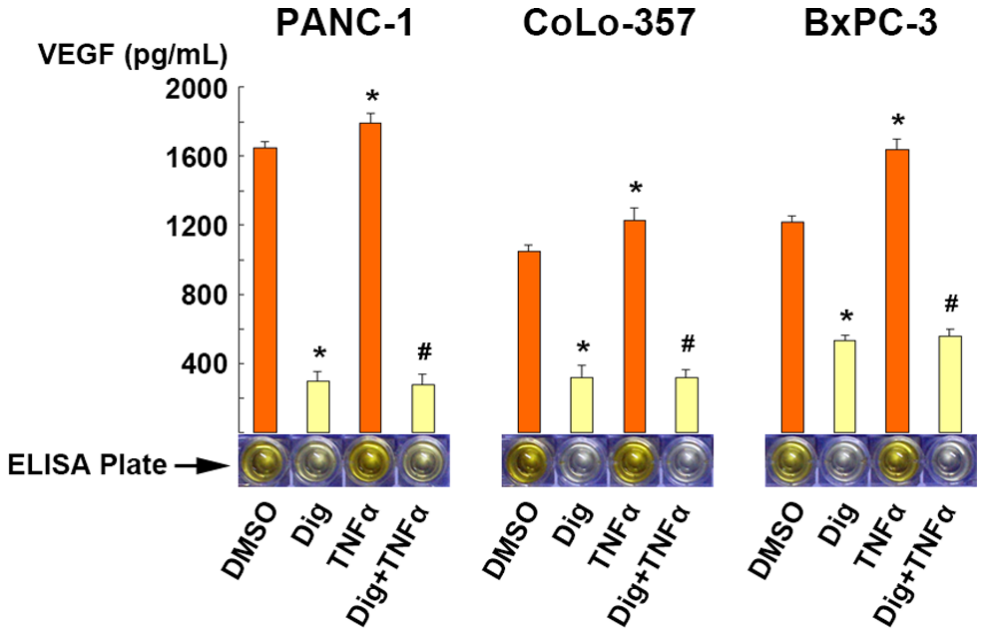
Digitoflavone suppressed VEGF secretion in pancreatic cancer cells. Representative data were shown from three independent experiments with identical results. VEGF was expressed as a picogram of VEGF protein per milliliter medium and per 10^5^ cells. *P<0.05 comparing to non-treated control; and #*P*<0.05 comparing to the group with TNFα only.

VEGF which is actively secreted from hypoxic tumor cells could trigger tumor angiogenesis. Reducing VEGF weakens its ability to stimulate tumor angiogenesis. Therefore we examined the effect of digitoflavone on VEGF secretion from the pancreatic carcinoma cells by using ELISA analysis. The results indicated that digitoflavone treatment for 24 h decreased VEGF secretion compared with the vehicle control group (*P*<0.05). Stimulation with TNFα increased VEGF secretion compared with the vehicle control group (*P*<0.05). However, pre-treatment with digitoflavone blocked the stimulation effect of TNFα (Fig. 8).

## Discussion

Pancreatic adenocarcinoma is an aggressive and highly lethal malignancy. Currently, gemcitabine is commonly used in patients with pancreatic cancer. However, the life expectancy of pancreatic cancer patients remains poor. Tsutom has reported that intratumoral injection of recombinant human TNFα inoperable cases of pancreatic cancer brought about regression of the tumor or a decrease in tumor markers [32]. However, a complete response had not been achieved in any of these cases, and the overall outcome was not sufficient, possibly because cytoprotecting factors such as enTNF and MnSOD are abundant in pancreatic cancer cells [33], or because TNF receptors were hardly expressed. This refractoriness to TNFα may be overcome by combination with a low cytotoxic compound, which can sensitize the effect of TNFα.

Digitoflavone is a common plant flavonoid which possesses anticancer properties that were demonstrated by previous studies [29]. Digitoflavone can be found in a large quantity of plants and foods, including beets, cabbage, cauliflower, celery, green pepper, perilla leaf, olive oil, and tea [34]. In cellular studies, digitoflavone has been found to possess anti-oxidant, anti-inflammatory/anti-allergic, anti-tumorigenic, and radical action [35], [36], [37]. Digitoflavone was reported to inhibit the development of a series of solid tumors [38], [39], [40], [41], [42], [43], [44], [45], [46], [47]. In this study, we provided evidence that digitoflavone sensitizes TNFα-induced apoptosis in human pancreatic cancer cells. Such sensitization is achieved via its inhibitory effect on NF-κB activation, which in turn results in reduced expression of antiapoptotic NF-κB target genes. Data from this study revealed a novel function of digitoflavone and enhance the value of digitoflavone as a useful anticancer agent.

NF-κB activation leads to the expression of genes that are involved in the proliferation and metastasis of cancer. In this report, we showed that digitoflavone inhibits the expression of cyclin D1, which is regulated by NF-κB. In addition, our results indicate that digitoflavone downregulates the expression of COX-2, MMP-9, and VEGF which are all regulated by NF-κB. These results further implied that digitoflavone exercised its anticancer properties through NF-κB inhibition. NF-κB regulated the expression of Mcl-1, Bcl-2, Bcl-X_L_, c-IAP1, c-IAP2, FLIP, and survivin, and their overexpression in numerous tumors has been linked to tumor cell survival, chemoresistance, and radioresistance. Our results indicate that digitoflavone treatment downregulates all these gene products. Digitoflavone has been shown to inhibit the growth of wide variety of tumor cells such as leukemic cells and non-small-cell lung carcinoma cells [30]. This growth inhibition may be mediated through downregulation of various genes. Downregulation of various antiapoptotic gene products by digitoflavone also sensitized the cells to the apoptotic effects of TNFα. Further studies have shown that digitoflavone had a good binding potency to the ATP binding site of IKK, which demonstrated that digitoflavone very likely inhibited NF-κB pathway through inhibition of IKK. Digitoflavone could active JNK and overexpression of p65 prevented digitoflavone-induced JNK activation. Dig might be a novel drug to provide a continuous blockade of the feed-back inhibitory mechanism by JNK-induced NF-kB activation. This may be the mechanism why digitoflavone can sensitize TNFα. Of course, more experimental verification including *in vivo* study was needed.

## References

[1] Jemal A, Tiwari RC, Murray T, Ghafoor A, Samuels A, et al (2004) Cancer statistics, 2004. CA Cancer J Clin 54: 8–29. 14974761 10.3322/canjclin.54.1.8

[2] Gold EB, Goldin SB (1998) Epidemiology of and risk factors for pancreatic cancer. Surg Oncol Clin N Am 7: 67–91. 9443987

[3] Blaszkowsky L (1998) Treatment of advanced and metastatic pancreatic cancer. Front Biosci 3: E214–225. 9792894 10.2741/a380

[4] Burris HA, 3rd, Moore MJ, Andersen J, Green MR, Rothenberg ML, et al (1997) Improvements in survival and clinical benefit with gemcitabine as first-line therapy for patients with advanced pancreas cancer: a randomized trial. J Clin Oncol 15: 2403–2413. 9196156 10.1200/JCO.1997.15.6.2403

[5] Baldwin AS (2001) Control of oncogenesis and cancer therapy resistance by the transcription factor NF-kappaB. J Clin Invest 107: 241–246. 11160144 10.1172/JCI11991 PMC199203

[6] Wang CY, Cusack JC Jr, Liu R, Baldwin AS Jr (1999) Control of inducible chemoresistance: enhanced anti-tumor therapy through increased apoptosis by inhibition of NF-kappaB. Nat Med 5: 412–417. 10202930 10.1038/7410

[7] Bardeesy N, DePinho RA (2002) Pancreatic cancer biology and genetics. Nat Rev Cancer 2: 897–909. 12459728 10.1038/nrc949

[8] Fujioka S, Sclabas GM, Schmidt C, Frederick WA, Dong QG, et al (2003) Function of nuclear factor kappaB in pancreatic cancer metastasis. Clin Cancer Res 9: 346–354. 12538487

[9] Hu L, Shi Y, Hsu JH, Gera J, Van Ness B, et al (2003) Downstream effectors of oncogenic ras in multiple myeloma cells. Blood 101: 3126–3135. 12515720 10.1182/blood-2002-08-2640

[10] Mayo MW, Wang CY, Cogswell PC, Rogers-Graham KS, Lowe SW, et al (1997) Requirement of NF-kappaB activation to suppress p53-independent apoptosis induced by oncogenic Ras. Science 278: 1812–1815. 9388187 10.1126/science.278.5344.1812

[11] Li L, Aggarwal BB, Shishodia S, Abbruzzese J, Kurzrock R (2004) Nuclear factor-kappaB and IkappaB kinase are constitutively active in human pancreatic cells, and their down-regulation by curcumin (diferuloylmethane) is associated with the suppression of proliferation and the induction of apoptosis. Cancer 101: 2351–2362. 15476283 10.1002/cncr.20605

[12] Farrow B, Evers BM (2002) Inflammation and the development of pancreatic cancer. Surg Oncol 10: 153–169. 12020670 10.1016/s0960-7404(02)00015-4

[13] Yamamoto Y, Gaynor RB (2001) Therapeutic potential of inhibition of the NF-kappaB pathway in the treatment of inflammation and cancer. J Clin Invest 107: 135–142. 11160126 10.1172/JCI11914 PMC199180

[14] Chen S, Fribley A, Wang CY (2002) Potentiation of tumor necrosis factor-mediated apoptosis of oral squamous cell carcinoma cells by adenovirus-mediated gene transfer of NF-kappaB inhibitor. J Dent Res 81: 98–102. 11827262

[15] Wang CY, Mayo MW, Baldwin AS Jr (1996) TNF- and cancer therapy-induced apoptosis: potentiation by inhibition of NF-kappaB. Science 274: 784–787. 8864119 10.1126/science.274.5288.784

[16] Aggarwal BB (2003) Signalling pathways of the TNF superfamily: a double-edged sword. Nat Rev Immunol 3: 745–756. 12949498 10.1038/nri1184

[17] Aggarwal BB (2004) Nuclear factor-kappaB: the enemy within. Cancer Cell 6: 203–208. 15380510 10.1016/j.ccr.2004.09.003

[18] Yamamoto Y, Gaynor RB (2001) Role of the NF-kappaB pathway in the pathogenesis of human disease states. Curr Mol Med 1: 287–296. 11899077 10.2174/1566524013363816

[19] Aggarwal BB, Takada Y, Shishodia S, Gutierrez AM, Oommen OV, et al (2004) Nuclear transcription factor NF-kappa B: role in biology and medicine. Indian J Exp Biol 42: 341–353. 15088683

[20] Karin M, Cao Y, Greten FR, Li ZW (2002) NF-kappaB in cancer: from innocent bystander to major culprit. Nat Rev Cancer 2: 301–310. 12001991 10.1038/nrc780

[21] Garg A, Aggarwal BB (2002) Nuclear transcription factor-kappaB as a target for cancer drug development. Leukemia 16: 1053–1068. 12040437 10.1038/sj.leu.2402482

[22] Giri DK, Aggarwal BB (1998) Constitutive activation of NF-kappaB causes resistance to apoptosis in human cutaneous T cell lymphoma HuT-78 cells. Autocrine role of tumor necrosis factor and reactive oxygen intermediates. J Biol Chem 273: 14008–14014. 9593751 10.1074/jbc.273.22.14008

[23] Estrov Z, Manna SK, Harris D, Van Q, Estey EH, et al (1999) Phenylarsine oxide blocks interleukin-1beta-induced activation of the nuclear transcription factor NF-kappaB, inhibits proliferation, and induces apoptosis of acute myelogenous leukemia cells. Blood 94: 2844–2853. 10515888

[24] Bharti AC, Donato N, Singh S, Aggarwal BB (2003) Curcumin (diferuloylmethane) down-regulates the constitutive activation of nuclear factor-kappa B and IkappaBalpha kinase in human multiple myeloma cells, leading to suppression of proliferation and induction of apoptosis. Blood 101: 1053–1062. 12393461 10.1182/blood-2002-05-1320

[25] Estrov Z, Shishodia S, Faderl S, Harris D, Van Q, et al (2003) Resveratrol blocks interleukin-1beta-induced activation of the nuclear transcription factor NF-kappaB, inhibits proliferation, causes S-phase arrest, and induces apoptosis of acute myeloid leukemia cells. Blood 102: 987–995. 12689943 10.1182/blood-2002-11-3550

[26] Bharti AC, Shishodia S, Reuben JM, Weber D, Alexanian R, et al (2004) Nuclear factor-kappaB and STAT3 are constitutively active in CD138+ cells derived from multiple myeloma patients, and suppression of these transcription factors leads to apoptosis. Blood 103: 3175–3184. 15070700 10.1182/blood-2003-06-2151

[27] Shishodia S, Aggarwal BB (2004) Nuclear factor-kappaB: a friend or a foe in cancer? Biochem Pharmacol 68: 1071–1080. 15313403 10.1016/j.bcp.2004.04.026

[28] Shishodia S, Amin HM, Lai R, Aggarwal BB (2005) Curcumin (diferuloylmethane) inhibits constitutive NF-kappaB activation, induces G1/S arrest, suppresses proliferation, and induces apoptosis in mantle cell lymphoma. Biochem Pharmacol 70: 700–713. 16023083 10.1016/j.bcp.2005.04.043

[29] Lin Y, Shi R, Wang X, Shen HM (2008) Luteolin, a flavonoid with potential for cancer prevention and therapy. Curr Cancer Drug Targets 8: 634–646. 18991571 10.2174/156800908786241050 PMC2615542

[30] Cai X, Ye T, Liu C, Lu W, Lu M, et al (2011) Luteolin induced G2 phase cell cycle arrest and apoptosis on non-small cell lung cancer cells. Toxicol In Vitro 25: 1385–1391. 21601631 10.1016/j.tiv.2011.05.009

[31] Bonizzi G, Piette J, Merville MP, Bours V (1997) Distinct signal transduction pathways mediate nuclear factor-kappaB induction by IL-1beta in epithelial and lymphoid cells. J Immunol 159: 5264–5272. 9548465

[32] Watanabe N, Yamauchi N, Maeda M, Neda H, Tsuji Y, et al (1994) Recombinant human tumor necrosis factor causes regression in patients with advanced malignancies. Oncology 51: 360–365. 8208522 10.1159/000227366

[33] Watanabe N, Tsuji N, Tsuji Y, Sasaki H, Okamoto T, et al (1996) Endogenous tumor necrosis factor inhibits the cytotoxicity of exogenous tumor necrosis factor and adriamycin in pancreatic carcinoma cells. Pancreas 13: 395–400. 8899800 10.1097/00006676-199611000-00009

[34] Sasaki N, Toda T, Kaneko T, Baba N, Matsuo M (2003) Protective effects of flavonoids on the cytotoxicity of linoleic acid hydroperoxide toward rat pheochromocytoma PC12 cells. Chem Biol Interact 145: 101–116. 12606158 10.1016/s0009-2797(02)00248-x

[35] Ashokkumar P, Sudhandiran G (2008) Protective role of luteolin on the status of lipid peroxidation and antioxidant defense against azoxymethane-induced experimental colon carcinogenesis. Biomed Pharmacother 62: 590–597. 18692983 10.1016/j.biopha.2008.06.031

[36] Brown JE, Khodr H, Hider RC, Rice-Evans CA (1998) Structural dependence of flavonoid interactions with Cu2+ ions: implications for their antioxidant properties. Biochem J 330 (Pt 3): 1173–1178. 10.1042/bj3301173 PMC1219258 9494082

[37] Galati G, Moridani MY, Chan TS, O’Brien PJ (2001) Peroxidative metabolism of apigenin and naringenin versus luteolin and quercetin: glutathione oxidation and conjugation. Free Radic Biol Med 30: 370–382. 11182292 10.1016/s0891-5849(00)00481-0

[38] Lim do Y, Jeong Y, Tyner AL, Park JH (2007) Induction of cell cycle arrest and apoptosis in HT-29 human colon cancer cells by the dietary compound luteolin. Am J Physiol Gastrointest Liver Physiol 292: G66–75. 16901994 10.1152/ajpgi.00248.2006

[39] Chang J, Hsu Y, Kuo P, Kuo Y, Chiang L, et al (2005) Increase of Bax/Bcl-XL ratio and arrest of cell cycle by luteolin in immortalized human hepatoma cell line. Life Sciences 76: 1883–1893. 15698865 10.1016/j.lfs.2004.11.003

[40] Lee HJ, Wang CJ, Kuo HC, Chou FP, Jean LF, et al (2005) Induction apoptosis of luteolin in human hepatoma HepG2 cells involving mitochondria translocation of Bax/Bak and activation of JNK. Toxicology and Applied Pharmacology 203: 124–131. 15710173 10.1016/j.taap.2004.08.004

[41] Lee WJ, Wu LF, Chen WK, Wang CJ, Tseng TH (2006) Inhibitory effect of luteolin on hepatocyte growth factor/scatter factor-induced HepG2 cell invasion involving both MAPK/ERKs and PI3K-Akt pathways. Chemico-Biological Interactions 160: 123–133. 16458870 10.1016/j.cbi.2006.01.002

[42] Plaumann B, Fritsche M, Rimpler H, Brandner G, Hess RD (1996) Flavonoids activate wild-type p53. Oncogene 13: 1605–1614. 8895505

[43] Yee SB, Lee JH, Chung HY, Im KS, Bae SJ, et al (2003) Inhibitory effects of luteolin isolated from Ixeris sonchifolia Hance on the proliferation of HepG2 human hepatocellular carcinoma cells. Archives of Pharmacal Research 26: 151–156. 12643593 10.1007/BF02976662

[44] Zhang Q, Zhao XH, Wang ZJ (2008) Flavones and flavonols exert cytotoxic effects on a human oesophageal adenocarcinoma cell line (OE33) by causing G2/M arrest and inducing apoptosis. Food and Chemical Toxicology 46: 2042–2053. 18331776 10.1016/j.fct.2008.01.049

[45] Schutte ME, Boersma MG, Verhallen DA, Groten JP, Rietjens IM (2008) Effects of flavonoid mixtures on the transport of 2-amino-1-methyl-6-phenylimidazo[4,5-b]pyridine (PhIP) through Caco-2 monolayers: an in vitro and kinetic modeling approach to predict the combined effects on transporter inhibition. Food and Chemical Toxicology 46: 557–566. 17935851 10.1016/j.fct.2007.08.038

[46] Yang SF, Yang WE, Chang HR, Chu SC, Hsieh YS (2008) Luteolin induces apoptosis in oral squamous cancer cells. Journal of Dental Research 87: 401–406. 18362328 10.1177/154405910808700413

[47] Xavier CP, Lima CF, Preto A, Seruca R, Fernandes-Ferreira M, et al (2009) Luteolin, quercetin and ursolic acid are potent inhibitors of proliferation and inducers of apoptosis in both KRAS and BRAF mutated human colorectal cancer cells. Cancer Letters 281: 162–170. 19344998 10.1016/j.canlet.2009.02.041

