# Correction: Digitoflavone Inhibits IκBα Kinase and Enhances Apoptosis Induced by TNFα through Downregulation of Expression of Nuclear Factor κB-Regulated Gene Products in Human Pancreatic Cancer Cells

**DOI:** 10.1371/annotation/1725f581-eff6-4a1d-89d4-78cf8213c3bb

**Published:** 2013-10-24

**Authors:** Xueting Cai, Wuguang Lu, Yang Yang, Jie Yang, Juan Ye, Zhenhua Gu, Chunping Hu, Xiaoning Wang, Peng Cao

A funding organization and grant were incorrectly included in the Funding Statement. The Funding Statement should read: "This work was supported by the National Natural Science Foundation of China (Nos. 81274150, 81374018, 81202967, and 30873410) and Jiangsu Province's Outstanding Leader Program of Traditional Chinese Medicine. The funders had no role in study design, data collection and analysis, decision to publish, or preparation of the manuscript." 

